# Container-Based Clinical Solutions for Portable and Reproducible Image Analysis

**DOI:** 10.1007/s10278-018-0089-4

**Published:** 2018-05-08

**Authors:** Jordan Matelsky, Gregory Kiar, Erik Johnson, Corban Rivera, Michael Toma, William Gray-Roncal

**Affiliations:** 1 Applied Physics Laboratory, Johns Hopkins University, 11100 Johns Hopkins Road, Laurel, MD 20723-6099 USA; 2 McGill University, Montreal, QC Canada

**Keywords:** Containers, Reproducibility, Docker, Singularity, Medical-imaging

## Abstract

Medical imaging analysis depends on the reproducibility of complex computation. Linux containers enable the abstraction, installation, and configuration of environments so that software can be both distributed in self-contained images and used repeatably by tool consumers. While several initiatives in neuroimaging have adopted approaches for creating and sharing more reliable scientific methods and findings, Linux containers are not yet mainstream in clinical settings. We explore related technologies and their efficacy in this setting, highlight important shortcomings, demonstrate a simple use-case, and endorse the use of Linux containers for medical image analysis.

## Introduction

The evolution of complex imaging methods, coupled with reductions in the cost of data storage, have led to increasingly large data in the clinical imaging community. In parallel, computational methods to analyze these images have grown more sophisticated and nuanced. While these new techniques represent exciting steps forward in state-of-the-art medical image processing, they also expose the field to new operational vulnerabilities: researchers must take special care to precisely emulate the computational environment and configuration of an algorithm developer, or risk the possibility of incorrect results. As neural networks and other complex analyses grow in popularity, ensuring that analyses are *reproducible* and *repeatable* has taken on new importance.

Even well-designed and highly compatible software can incur issues when researchers’ computers do not perfectly match software developers’ operating system, configuration, or installed libraries. Resulting findings may therefore fail to truly enable experiment reproducibility.

Invented in 2008, *Linux containers* provide a way to “freeze” an environment in a simple package, enabling serialization and redeployment [1, 2]. However, Linux containers in their basic form remain relatively esoteric and difficult to use for the majority of non-expert users. For this reason, many software solutions exist to simplify the process of tool or environment containerization. *Containerization* enables the distribution of software bundled alongside its required packages and libraries, increasing the portability of software and the ability to reproduce scientific analyses. *Docker* and *Singularity* are two emerging platforms which provide user-friendly avenues for containerization and serve distinct communities based upon differences in core features such as data security and accessibility [3, 4].

## Background

**Fig. 1:**
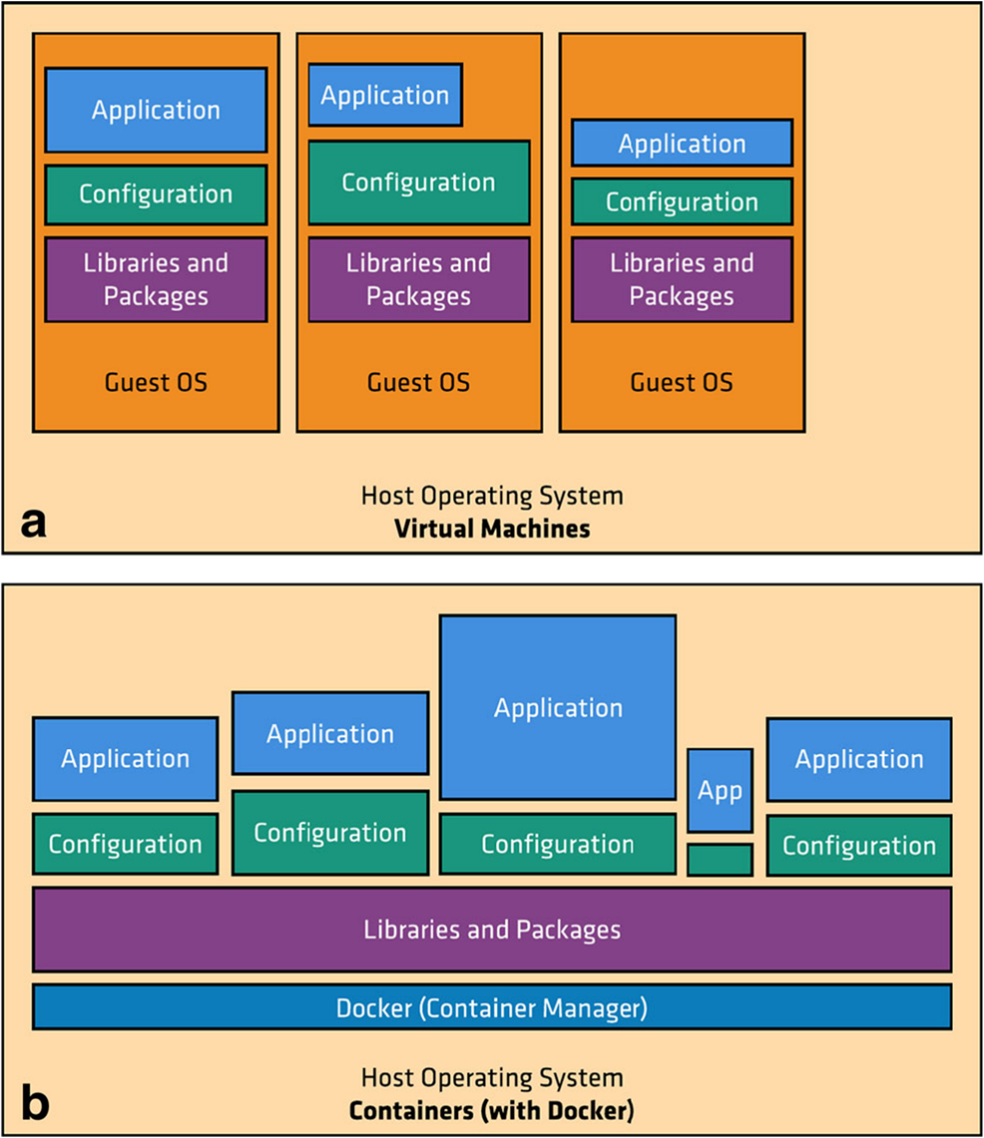
**a** Virtual machines each require their own guest operating system (OS), libraries, and configuration. The VMs pictured above have pre-allocated sizes and use hard drive space and RAM even when idle. **b** In contrast, containers do not require a guest operating system, can share libraries, and only the resources needed for a particular analysis

Researchers have adopted a variety of strategies in order to homogenize compute platforms to enable reproducible software-based analyses. Some tools, such as Python *virtual environments* and *Conda*, modify the local environment, but cannot provide cross-platform reproducibility or extend beyond specific language barriers [5]. *Virtual machines* (VMs) are one form of portable cross-platform compute environments that enable highly reproducible compute environments regardless of host operating system or environment [6]. Some cloud compute providers such as Amazon Web Services (AWS) offer their own versions of these—the most popular being Amazon Machine Images (AMIs)—which are commonly used and distributed, but only available on specific vendors’ computing resources [7]. Unfortunately, virtual machines are costly in terms of resource usage. VMs must contain a full operating system which leads to large amounts of overhead, even for simple analytical pipelines [8]. Because VMs are fully virtualized computers, they must be “booted up,” which dramatically increases the amount of time required in order to perform an analysis. Furthermore, VMs most often require that resources such as RAM or hard-drive space are fully devoted and allocated prior to the first boot up. This means that it is increasingly difficult to run multiple virtual machines on the same host machine, even when the VMs are idle (Fig. 1a).

*Containers* are another tool commonly tapped for the purposes of resource abstraction. Like virtual machines, containers enable rapid deployment of tools across various infrastructures, and they shift the burden of installation to tool developers rather than users. These environments can be deployed consistently across computational infrastructures and are useful for debugging and understanding tools. Unlike virtual machines, containers do not require resource allocation at creation time: Rather, resources are *shared* in real time with the host system (Fig. 1b).

Containerized software has seen wide adoption in the software development community, for both commercial (AWS [9], Google Gloud [10], Azure [11], etc.) and academic platforms (SIC [12], CBRAIN [13], Virtual Imaging Platform [14]). These groups have adopted containers as a way to repeatably install and deploy tools. Adoption efforts by neuroimaging groups have helped to bring containerization into the forefront of the medical imaging analysis consciousness. In particular, container-based neuroimaging pipelines such as MRI analysis serve as an efficient vehicle for sharing code and analyses between development environments and higher-performance production workloads.

## Container Managers

Both Singularity and Docker provide self-contained environments which enable command-line or graphical execution of tools or services. Each of these systems leverages the host system’s kernel for performing tasks, meaning that while the encapsulated tools are identical, the underlying execution may differ slightly across computational infrastructures.

### Docker

Docker is the most widely used container management system because it is installable on all major operating systems and its configuration language is mature, simple, and well documented [15]. This makes Docker a highly accessible solution for developing and wrapping tools. Docker’s major limitation is that of security: The software *inside* the container can be granted elevated permissions, in which case malicious software could access or manipulate the host system. This is often not an issue on personal workstations or commercial clouds, as the host systems are small, isolated from one another, and contain little persistent data, and so we recommend Docker for these settings [15] (Table 1).

**Table 1:** A comparison of the basic features of Docker and Singularity

Feature	Docker	Singularity
Secure	$\times $	✓
Scalable	✓	✓
Cross-compatible	$\times $	✓
Supports all major OSes	✓	$\times $
Accessible documentation	✓	$\times $

Each of these platforms provides a powerful lightweight solution to reliable, portable computing. Aside from this, Docker benefits from a large user community, rich documentation, and the ability to be deployed easily on all major operating systems (including Windows), whereas Singularity is less mature in these areas. The differentiating strength of Singularity lies in its ability to be deployed securely across shared high-performance computing infrastructures, preserving user access restrictions, whereas Docker is not suitable for these applications. Singularity is also capable of converting Docker images, lending itself to the popular use-case of being a deployment engine for containers developed locally through Docker

### Singularity

Singularity’s major contribution addresses Docker’s security shortcomings. Singularity restricts permissions within a container to those of the user launching the task. Singularity also enables HIPAA-compliant access-logging and differential privacy [16]. For this reason, Singularity is the platform of choice for many high-performance or high-security compute environments.

Singularity has seen less adoption due to its more recent introduction and inability to run on Windows or OSX systems. It therefore has a smaller support community and base of documentation than Docker, but enables the importing and conversion of most Docker containers, which reduces the need for developers to become intimately familiar with both frameworks.

## Use-Cases for Containerization

One common pitfall when deploying software in a controlled environment is that of operating system versioning. Systems are often “frozen” to older versions of an operating system in order to either maintain compatibility with mission-critical software or pause at a known stable or secure version of a supported OS. This becomes a problem when new research products require more modern libraries than the underlying OS natively supports.

This problem is solved by installing a container manager, such as Docker, on the older platform. Barring extenuating concerns, including inability to install such software on the older platform or numerical precision fragility (discussed below in *Risks and Limitations*), containerized software will deliver the same results on out-of-date host operating systems as it would on modern ones, without incurring the additional labor and cost of upgrading resources.

### Workflow Optimization

A common convention is to run atomic steps of an analysis in separate containers. That is, rather than installing *all* software in one large container, each container holds only a single step. For an imaging pipeline, this might mean that a color correction step lives in the first container, an alignment step lives in the second, and an image processing step such as lesion detection lives in the third.

Between steps, byproducts are stored in *volumes*, or mountable directories that can be shared with the host OS so that files persist after the container is destroyed [17]. This enables complex error handling or retry behavior: if the first step of an analysis fails, no other containers need to be run; if the second step fails, the results of the first step do not need to be recalculated. Furthermore, multiple algorithms with different requirements can be integrated by building Docker containers with different installation dependencies.

This stepwise paradigm is so common that there are many existing pipelining systems that fulfill just this need [18–21].

These tools, in conjunction with containers, enable detailed logging and rich capturing of execution outputs and progress. This provides reliable work histories for reproducible analysis and debugging.

### Integrating Docker into a Clinical Workflow

#### The Project

In a hypothetical workflow, a scientist may want to normalize the contrast of an MRI scan in order to increase contrast. A developer on the team writes a Python script called normalize_scan.py that takes three arguments from the command-line:

$ python normalize_scan.py [DICOM file] [min value] [max value]

and outputs the same file with the suffix -normalized, with the data remapped to the minimum/maximum values provided.

These scientists work on a shared host machine, and the software dependencies for this tool may conflict with those used by other labs.

#### Writing a Dockerfile

If this tool were run on a new system, the scientists would get the Python Module Not Found error telling them that their environment was not properly configured with the libraries required to run their script. Luckily, the software developer provided a requirements.txt which lists all required Python libraries.

In this simple case, the scientists can simply read the imports from the Python file and install them, but this method is less tractable for applications which depend upon code written in multiple languages, or those developed by multiple disparate teams.

The team decides to execute their code inside a Docker container in order to prevent version conflicts and to enable workflow reproducibility.

Leveraging prior work by other open-source developers, the scientists can use an existing container that has certain libraries pre-installed. For the purpose of illustration, we’ll use pydicom/dicom [22]. (In clinical environments, it may be wiser to build the container from scratch in order to have full control over the execution environment and eliminate unneeded dependencies.)

To indicate this to the image-building software packaged with Docker, the Dockerfile will begin with the line:

FROM pydicom/dicom

It is considered best practice to include the name of the maintainer of this Dockerfile, in case future users have questions:

LABEL maintainer="Your Name<your@email.com>"

The next step is to copy the Python script to the image:

ADD ./normalize_scan.py /src/normalize_scan.py

...as well as the requirements.txt file. The following line executes pip, the Python package manager, in order to install the libraries listed in this file.

ADD ./requirements.txt /src/requirements.txtRUN pip install -r /src/requirements.txt

Finally, the Dockerfile indicates to Docker what command it should run when the container starts by providing an “entrypoint.” In this example, all of the scans will be remapped to the [0..100] range. One could allow for user- defined inputs using environment variables, as we do in code provided at https://github.com/jdi-matelsky-et-al-2018/.

ENTRYPOINT python3 /src/normalize_scan.py/infile.dcm /mnt/vout/ 0 100

This code makes the assumption that our scan exists at the path /infile.dcm. This is a safe assumption, as we’ll discuss in Section 2.

#### Building the Image

Next, the Docker *image* can be built. The image may take some time to build while it downloads and installs packages, but this only needs to be done when changes are made to the contents of the image: Afterward, Docker *containers* can be launched quickly based on this image template.

To build the container and tag it with the name of remap-dicom, the scientists will run the following command:

docker build -t username/dicom-remap .

where the “username” field above may correspond to the scientist’s account on Docker Hub, a sharing platform for Docker images [23]. This enables the image builder to (optionally) “push” the image and download it on other machines or, if shared publicly, enable others to use it.

This command will retrieve the latest version of the pydicom/dicom image from Docker Hub and will then run the commands from the above Dockerfile in order, caching the results of each step so that successive builds are faster and more efficient.

#### Building a Singularity Image

If the scientists wish for their tool to be available for others who are using platforms which require Singularity rather than Docker, they can easily leverage the above work in order to create a Singularity image hosted instead on Singularity Hub. There are two common approaches for this: The first is through the docker2singularity utility [24], and the second is by creating a Singularity file. The former method is an open-source tool which walks the user through the conversion of Docker images to Singularity images. The latter is a much shorter equivalent of the Dockerfile created above but it relies on pushing the Docker image to Docker Hub. If the image is accessible online, this file can contain just the two lines below:

Bootstrap: docker From: username/dicom-remap

#### Running Docker Containers

The final step is running the container. To do this, the scientist navigates into the directory with the image file they wish to normalize, called myfile.dcm and runs the following command:

mkdir ./out docker run -v $(pwd)/myfile.dcm:/infile.dcm -v $(pwd)/out:/mnt/vout dicom-remap

The -v syntax indicates a Docker *volume*: This is a mapping from the host’s filesystem to the private Docker filesystem. Because actions inside the container do not affect the host environment, the scientist must deliberately allow the container to make changes to only specific files. *Two* volumes are mounted by the command above. The first is the target file, myfile.dcm, which is *mounted to* the /infile.dcm location (referenced earlier). Simply, this allows the container to access this under the alias of /infile.dcm.

The second mounted volume is the output directory. An empty directory on the host file system is mounted, $(pwd)/out, to the directory /mnt/vout/ in the container. When a file is placed in the container’s /mnt/vout/ directory, it will appear in the host machine’s $(pwd)/out/ directory.

By mounting these locations rather than a full working directory, data access is restricted within the container. Deliberate and careful use of volumes improves container security.

## Risks and Limitations

While containers simplify many aspects of dependency management and analysis reproducibility, this technology requires that a user manage container installation and execution. This may provide a barrier to entry in cases where potential users lack the necessary training. End-user container management applications are typically straightforward, and writing new recipes (i.e, Dockerfiles) may be unfamiliar to some researchers. Despite this, we observe that existing tutorials and seed containers make this straightforward for most users.

Containers may *reduce* the accessibility of container-based tools when users are restricted to environments that do not support container management systems (e.g. old operating systems or platforms that dramatically restrict user permissions). This can be mitigated by system administrators aiming to provide up-to-date environments for researchers.

Because container use abstracts operating system selection and installation processes from tool users, the tool user has less control over the eventual execution environment. This can lead to incompatibilities that are not obvious to the end user. One particularly subtle example of this is the practice of pointing to the “latest” version of libraries when installing. This makes the image brittle to changes in updates to its dependency libraries. If another user builds the image after a library has updated its latest-deployed version, the resultant image will differ from the author’s. Users can easily remedy this issue by explicitly specifying recommended versions of libraries or software when distributing images.

Recent research has suggested that the use of containers may obfuscate the origin of numerical instabilities running inside the container. These differences may only become apparent when deploying software across multiple operating systems using the same data and comparing the results. Several recent studies (in submission, OHBM 2018 [25]) have demonstrated significant differences in MRI brain segmentation when running the popular Pre-FreeSurfer pipeline [26] between different operating systems and configurations. It is suspected that these differences between operating systems are the result of underlying numerical instabilities in the underlying algorithms, and numerical libraries may be performing different approximations or handling of these exceptions.

We assert that these issues are irrelevant for the majority of users and suggest exercising caution and validating results when deploying software both when using and when *not* using containers.

## Discussion

Because clinical and medical sciences are increasingly reliant on complex algorithms in order to derive insight from data, analysis repeatability or reproducibility should be considered a high priority (see [27] for a review of these terms and their various definitions). Many medical image analysis developers are beginning to provide their tools in containerized environments, enabling portable, repeatable analyses. Spearheading this containerization effort are Singularity and Docker, two platforms that simplify the process of developing and deploying container-based applications. These systems hold the promise of dramatically reducing the complexity of configuring and installing medical imaging analysis tools and pipelines.

These tools have been deployed in a wide range of infrastructures, including privacy-sensitive settings such as the XSEDE super computing cluster in the USA [28], Compute Canada [29], and other commercial clouds [9]. While adoption in clinical research environments is still low, these platforms can serve as valuable tools when bridging the gap between research and clinical settings.
